# Forimtamig: a potent GPRC5D-targeted bispecific antibody in multiple myeloma

**DOI:** 10.1097/MS9.0000000000004952

**Published:** 2026-04-20

**Authors:** Hafsa Ali, Hiba Hilal, Maliha Gul, Maham Asad, Mohammed Hammad Jaber Amin

**Affiliations:** a Department of Medicine, Sindh Medical College, Jinnah Sindh Medical University, Karachi, Pakistan; b Department of Medicine, Karachi Medical & Dental College, North Nazimabad, Karachi, Pakistan; c Department of Medicine, Khyber Girls Medical College, Hayatabad, Peshawar, Pakistan; d Department of Medicine, Faculty of Medicine, Alzaiem Alazhari University, Khartoum, Sudan

**Keywords:** Multiple myeloma, forimtamig, GPRC5D-targeting TCB, N-terminal region

## Abstract

Multiple myeloma is a malignancy of clonal plasma cells characterized by progressive bone marrow infiltration, commonly presenting with anemia, bone pain, hypercalcemia, and renal dysfunction. T-cell bispecific antibodies (TCBs) have emerged as an effective therapeutic option in relapsed and refractory multiple myeloma, with G-coupled protein receptor, family C, group 5, member D (GPRC5D) representing a promising target due to its high expression in myeloma cells and limited presence in normal tissues. Forimtamig is a next-generation GPRC5D-targeting TCB with a novel 2:1 binding format – bivalently engaging GPRC5D and monovalently binding CD3ε on T cells. This structural advantage enhances avidity, stabilizes immunological synapses, and enables potent T-cell activation and cytotoxicity, even against low-antigen-expressing cells in immunosuppressive bone marrow environments. Preclinical studies show that forimtamig outperforms earlier 1 + 1 TCB formats by 50–200-fold in cell-killing efficiency. When combined with standard agents such as daratumumab, pomalidomide, or cereblon E3 ligase modulatory drugs, forimtamig has shown improved tumor clearance and reduced relapse rates. Its design also minimizes tumor escape by targeting the N-terminal region of GPRC5D. Currently undergoing phase 1 trials, forimtamig is being evaluated both as monotherapy and in combination regimens. Its high potency, favorable safety, and durable immune engagement make it a promising candidate in the evolving treatment landscape of multiple myeloma.

## Introduction

Multiple myeloma, a clonal B-cell malignancy, is most often described by the abnormal accumulation of mutated plasma cells in the bone marrow^[^1^]^. It is the second leading cause of cancer-related mortality worldwide, accounting for 10% of all blood-related malignancies^[^2^]^. Risk factors include age, family history, chromosomal abnormalities, and existing conditions like monoclonal gammopathy of undetermined significance. Patients present with complaints of bone pain, anemia, kidney problems, and increased levels of calcium. One effective strategy in the treatment of multiple myeloma is using T cell–specific antibodies (TCBs), which recruit and activate T cells directly against cancerous cells. A particularly interesting target is a G-coupled protein receptor, family C, group 5, member D (GPRC5D), highly expressed in myeloma cells^[^3,4^]^. It is a very promising target because of its minimal expression in normal body cells^[^4^]^. These therapies have shown high response rates, strong and lasting effects, favorable safety profiles, and are readily available. Three TCBs have recently been approved (teclistamab, elranatamab, and talquetamab). They exhibit a 1 + 1 mechanism^[^5^]^, meaning one part binds to the cancer cell receptor while the other binds to CD3ε of the T cell. However, these drugs have faced challenges like short-lived response and chances of relapse due to target antigen reduction^[^6^]^. Moreover, when administered, telquetamad showed specific adverse effects such as cytokine release syndrome, skin-related events, and dysgeusia^[^7,8^]^. Thus, next-generation therapies with greater potency and durability are needed. Forimtamig is a novel and more potent GPRC5D-TCB. It uses a 2 + 1 binding format, enabling bivalent binding to GPRC5D and monovalent to CD3 of T cells, allowing more affinity than the 1 + 1 format for an effective immune response and more potent recruitment of T cells. Moreover, integration with the standard-of-care (SOC) agents showed enhanced response, while a more progression-free survival was observed when combined with cereblon E3 ligase modulatory drugs (CELMoDs).^[^2^]^

## Forimtamig and its mechanism of action

Forimtamig is a next-generation bispecific antibody specifically engineered to target GPRC5D, a surface protein primarily expressed on plasma cells. It has a unique 2:1 structure, i.e., two identical Fab arms bind to the N-terminal end of GPRC5D, while the third arm targets CD3ε on T cells.

The high avidity results from the dual binding to GPRC5D, which means that it attaches to the cancerous cell with exceptional strength and stability. This powerful binding acts as an anchor and is vital to immune activation as it catalyzes the formation of stable immunological synapses, which establish a strong bond between T cells and tumor cells. Moreover, by enabling increased regulation of CD69 and CD25, along with secretion of granzyme-B (GZB), interferon gamma (IFN-γ), and interleukin-2 (IL-2), it potently activates CD8^+^ and CD4^+^ T cells^[^2^]^.

An immunological synapse is a zone where a T cell and a tumor cell connect. Forimtamig promotes this by slowing T-cell movement and increasing the duration of contact between the T cell and the myeloma cell. This firm and prolonged contact allows the T cell enough time to organize its internal structures (particularly the cytoskeleton) to direct the release of toxic proteins such as perforin and granzymes. These molecules are emitted precisely when and where they are needed. They work by creating holes in the tumor cell membrane and initiating the process of cell death from the inside. The longer and more stable the synapse, the more effective the killing. Forimtamig has demonstrated remarkable potency in lab and animal studies, including the myeloma cells with very low GPRC5D expression. It surpassed older formats by over 50–200 times in cell-killing efficiency. It also worked well in the bone marrow environment, where many treatments struggle due to immunosuppression. There, forimtamig still activated T-cells, leading to a marked decrease in malignant plasma cells^[^2^]^.

Significantly, by targeting the N-terminal, a region unlikely to be lost during disease progression, Forimtamig highly reduces the chances of tumor escape. Its strong, sustained action makes it a promising treatment option, especially in combination therapies for deep and durable responses^[^2^]^.

## Clinical implications and future directions

Combining forimtamig with standard treatments such as anti-CD38 antibodies, immunomodulatory drugs, and proteasome inhibitors has enhanced the depth and duration of patient responses. When combined with SOC options such as daratumumab, pomalidomide, or both, forimtamig triggered the death of 34% of malignant myeloma cells. Forimtamig, with a 1 + 1 BCMA TCB, can halt tumor relapse and promote a lasting response. Additionally, incorporating forimtamig with therapeutic agents like BCMA TCBs and CELMoDs has shown effectiveness in preventing relapses in tumors that lack GPRC5D. As of now, forimtamig is undergoing evaluation in phase 1 clinical trials for patients with relapsed and refractory multiple myeloma, both as a standalone treatment and in combination with other therapies^[^2^]^. Future studies need to establish the efficacy of forimtamig in combination with teclistamab, daratumumab, pomalidomide, an anti-PD-1 antibody, carfilzomib, and lenalidomide with longer follow-up. Along with these trials, therapies such as CAR-natural killer cells, bispecific natural killer cell engagers, and antibody-drug conjugates may further improve treatment outcomes. Taking advantage of GPRC5d-targeting T-cell–redirecting agents in multiple myeloma treatment is important. Further analysis is required to establish the optimal sequence for T-cell–redirecting therapies that could bring positive outcomes^[^9^]^.

## Conclusion

Forimtamig shows a promising advancement in treating relapsed and refractory multiple myeloma. It offers good potency, a better safety profile, and immune activation compared to existing T-cell bispecific antibodies. Its 2 + 1 binding format is unique and offers stronger binding of GPRC5D and efficient T-cell engagement, even in low-antigen-expressing tumor cells and immunosuppressive bone marrow environments. When used in combination with SOC agents and newer therapies like CELMoDs or BCMA TCBs, forimtamig can potentially prolong prognosis and therapeutic responses simultaneously, reducing the risk of relapse. Ongoing trials and clinical research will play a key role in establishing its ideal role and position in the evolving multiple myeloma treatment landscape.

## Data Availability

Data sharing does not apply to this article as no new data were created or analyzed in this study.
